# Densitometry Versus Bioimpedance for Modeling Vitamin D–Endocrine and Metabolic Associations in Pediatric Obesity: A Cross-Sectional Parallel-Modality Analysis

**DOI:** 10.3390/nu18050750

**Published:** 2026-02-26

**Authors:** Elżbieta Jakubowska-Pietkiewicz, Jędrzej Chrzanowski, Elżbieta Woźniak

**Affiliations:** 1 Department of Pediatrics, Newborn Pathology and Bone Metabolic Diseases, University of Lodz, 91-738 Lodz, Poland; elzbieta.wozniak@umed.lodz.pl; 2 Department of Biostatistics and Translational Medicine, Medical University of Lodz, 92-215 Lodz, Poland; jedrzej.chrzanowski@umed.lodz.pl

**Keywords:** pediatric obesity, vitamin D, body composition, parallel studies

## Abstract

**Background/Objectives**: It has been previously shown that bioimpedance assessment (BIA) systematically underestimates adiposity compared to densitometry analysis (DXA), though the methods correlate strongly. However, whether DXA outperforms BIA for physiology modeling—using vitamin D as a sentinel signal—remains uncertain. We compared DXA and BIA side-by-side to model (i) adiposity–25(OH)D associations, (ii) mediation-style links with metabolic outcomes, and the vitamin D–PTH–calcium axis. **Methods**: We performed a cross-sectional analysis of 165 children with simple obesity and no vitamin D prophylaxis collected between July 2022 and July 2025. We measured adiposity through DXA and BIA methods, laboratory 25(OH)D, and associated biochemical and clinical parameters: PTH, calcium, phosphate, glucose/insulin/HOMA-IR, lipids. Information on age, sex, and season was recorded and used to adjust for potential covariates. Parallel analyses included partial correlations, linear regression, mediation models, and Bland–Altman analysis for DXA–BIA agreement. **Results**: The cohort median age was 13 years; median 25(OH)D level was 21.9 ng/mL. DXA fat % exceeded BIA (46.6% vs. 36.7%). Univariately, 25(OH)D correlated inversely with adiposity (DXA rho = −0.16, BIA rho = −0.19), but adiposity was not a significant determinant of 25(OH)D after season/age adjustment with either modality. No mediation of vitamin D to metabolic associations via adiposity were detected. The vitamin D–PTH–calcium axis was robust across modalities. **Conclusions**: In children with established obesity, seasonal and age factors dominate 25(OH)D variability, while the adiposity contributes little within-group. Vitamin D shows endocrine but not metabolic associations, and within this homogenous pediatric obesity cohort, DXA does not outperform BIA for physiologic modeling.

## 1. Introduction

Obesity is a complex, chronic disease with a recurrent nature, resulting from a positive energy balance, which leads to excessive fat accumulation in the body. It is a global health problem with a multifactorial etiology (genetic, neurobiological, economic, and environmental). WHO data indicate that the prevalence of obesity worldwide more than doubled between 1990 and 2022, affecting 43% of people over 18 years of age. Unfortunately, in the population of children and adolescents aged 5–19, the rate of increase in obesity prevalence is similar. It is estimated that in 2022, obesity affected 8% of children and adolescents aged 5–19 years (approximately 160 million individuals) [1].

The diagnosis of obesity in children and adolescents, as in adults, is based on the assessment of body mass index (BMI), but in relation to age and gender, using percentile charts or z-scores. Anthropometric measurements of body weight and height and the BMI calculated on their basis are the simplest, most accessible, and cheapest method of diagnosing obesity. According to the position of the Polish Society for Childhood Obesity and other societies involved in the diagnosis and treatment of childhood obesity, in Poland, obesity is diagnosed in children when their BMI is greater than the 97th percentile. This is in line with the WHO guidelines [1,2]. However, it is acceptable to use the 95th percentile as a diagnostic criterion [3,4].

Body composition assessment using bioimpedance (BIA—bioimpedance analysis) and densitometry (DXA—densitometry dual-energy X-ray absorptiometry) can supplement the diagnosis of childhood obesity. However, scientific society guidelines emphasize that these methods are not necessary and their use is limited to selected clinical situations, scientific research, or specialist care. Electrical bioimpedance is a non-invasive and readily available method for estimating body composition, including the percentage of body fat and fat-free mass. Densitometry using the DXA method allows for precise assessment of body composition, including the amount and distribution of body fat and bone mineral density. This method is considered the gold standard in scientific research [5].

Methodologically, densitometry analysis (DXA) is a reference for the body composition, but bioimpedance (BIA) is more practical and widely deployed. Although BIA underestimates fat % with wide limits of agreement, correlations with DXA are high [6,7,8]. Importantly, limited absolute agreement does not necessarily imply poor inferential validity: if a method preserves relative between-subject differences, it may still yield similar estimates of associations with physiologic endpoints. Whether these differences affect the interpretation of clinical and physiological associations, specifically in the context of vitamin D- and obesity-related biology, remains unclear.

The impact of vitamin D on health results from its calcitropic and pleiotropic effects and, in addition to regulating calcium and phosphate metabolism, includes reducing cardiovascular risk and the incidence of cancer, infectious diseases, nervous system diseases, and diabetes [3,9,10,11]. However, data indicate that vitamin D deficiency affected 45% of the global population between 2000 and 2020 [9]. Vitamin D deficiency (VDD) is defined as a concentration of its hepatic metabolite, 25(OH)D, equal to or lower than 20 ng/mL, and the absolute minimum recommended concentration is 30 ng/mL. However, it depends on overall health and morbidity, and for obese people it should be at least 40 ng/mL [9]. Widespread vitamin D deficiency also affects the Polish population, and its deficiency is defined in the same way, with supplementation recommendations depending on age, body weight, and vitamin D intake. The recommendations point out that being overweight or obese are risk factors for vitamin D deficiency, and the recommended dose of cholecalciferol in overweight children and adolescents should be doubled compared to their peers with normal weight [11].

Serum 25(OH)D is generally lower with a greater adiposity, as indicated by DXA, BIA, BMI, and waist circumference metrics [12,13,14]. Mechanisms include adipose sequestration, volumetric dilution, and reduced hepatic 25-hydroxylase (CYP2R1) activity [15,16,17,18]. Observational links between vitamin D and insulin resistance or dyslipidemia often diminish after adiposity adjustment [19,20,21,22], whereas the vitamin D–PTH–calcium axis remains physiologically consistent, even in pediatric obesity, and BMD is typically preserved or elevated [23,24,25,26].

We therefore conducted a parallel-modality analysis in 165 children with obesity (no vitamin D supplementation) to test (1) whether adiposity–vitamin D associations differ by DXA vs. BIA; (2) whether adiposity mediates proposed vitamin D–metabolic links; and (3) whether the vitamin D–PTH–calcium signal is modality-independent, while accounting for season and age.

## 2. Materials and Methods

### 2.1. Design and Participants

This was a cross-sectional study of 165 children/adolescents with obesity (BMI ≥ 97th percentile) and no vitamin D prophylaxis. Demographics, anthropometry, body composition (DXA and BIA), and laboratory markers were extracted from clinical records. Season of sampling was categorized (reference: autumn). The inclusion criteria were a diagnosis of simple obesity according to the definition (BMI > 97th percentile), no vitamin D prophylaxis, complete medical documentation, and consent for the use of data in publication. The exclusion criteria were age below 6 years and above 17 years, syndromic obesity, endocrine disorders, and lack of complete medical documentation. Participants were consecutively recruited from children attending a tertiary outpatient clinic for non-syndromic (simple) obesity. Sample size reflected all eligible children during the study period with complete DXA, BIA, and laboratory data. No formal sample size calculation was performed, consistent with exploratory physiologic modeling studies.

The study was conducted in accordance with the Declaration of Helsinki and was approved by the Bioethics Committee of the Medical University of Lodz RNN/138/25/KE (15th April 2025). Written informed consent was obtained from parents or legal guardians, and assent was obtained from all children capable of providing it. Data were collected from clinical records between July 2022 and July 2025.

### 2.2. Measurements

The primary exposure was the adiposity measured in parallel by a dual-energy X-ray absorptiometry (DXA, Horizon WI Densitometer, Hologic, Belgium) on TBLH (total body less head) program and bioimpedance (BIA; Tanita MC 780 MAP, Tanita Europe B.V, The Netherlands). Our modeled signal of interest was serum 25-hydroxyvitamin D [25(OH)D, ng/mL], measured using the ELISA method. Other variables of interest included parathyroid hormone (PTH), serum total calcium, phosphate, fasting glucose and insulin with HOMA-IR, and the lipid profile (total cholesterol, LDL-cholesterol, HDL-cholesterol, and triglycerides) in accordance with commonly accepted methods. Age, sex, and date of sampling (for assessment of season following meteorological definition) were included as covariates in all multivariable analyses.

To reduce measurement bias, standardized protocols for DXA and BIA were used. Missing data were handled using pairwise complete-case analysis. Because data were derived from clinical records, selection bias is possible; however, consecutive sampling minimized this risk.

### 2.3. Statistical Analysis

Continuous variables were summarized as mean  ±  SD or median (IQR) according to distribution; categorical variables as counts and percentages. To assess the method agreement between DXA and BIA within individuals, we conducted Bland–Altman analysis to estimate the mean bias and dispersion. Next, we ran association models in parallel for each modality. Univariate Spearman correlations revealed an inverse association between the 25(OH)D and adiposity (DXA fat% vs. BIA fat%). Next, with multivariable ordinary least-squares (OLS) regressions, we modeled 25(OH)D as the dependent variable with adiposity, age, sex, and season as predictors to determine whether adiposity assessment modality altered their inference in context of vitamin D. Third, we examined the endocrine and the metabolic endpoints—PTH, calcium, phosphate, HOMA-IR, LDL, HDL, and triglycerides—using linear regression models that included the 25(OH)D with and without the adiposity to test whether vitamin D showed independent associations and whether any putative vitamin D effects were mediated by the adiposity. To evaluate whether the adiposity could account for the association between the 25(OH)D and the metabolic outcomes, we conducted ‘mediation-style’ regression analyses in a cross-sectional framework. Specifically, for each outcome (e.g., HOMA IR, lipids, or BMD), we estimated: (i) the association between the 25(OH)D and the adiposity (path a), (ii) the association between the adiposity and the outcome adjusted for the 25(OH)D (path b), and (iii) the total association between the 25(OH)D and the outcome (path c) and the direct association adjusted for the adiposity (path c′). Indirect effects were interpreted descriptively as a × b, with the primary goal of assessing the attenuation patterns rather than causal mediation. To satisfy model assumptions, we applied log-transformation where needed; conclusions remained unchanged and are reported on the primary scale for the interpretability, unless otherwise noted. We have complemented regressions with the residual-based partial correlations of the 25(OH)D with each endpoint, controlling for season alone and for season plus the adiposity (separately for DXA and BIA) to visualize the attenuation patterns.

Analyses were conducted in Python 3.13 using statsmodels and scipy and visualized with seaborn and matplotlib. Statistical significance was interpreted at α  =  0.05. As this study was exploratory but hypothesis-driven and our primary goal was comparative inference between the DXA- and the BIA-based models, we did not apply a formal multiple testing correction; instead, we interpreted *p*-values cautiously and emphasized effect sizes and consistency across modalities.

## 3. Results

### 3.1. Participant Characteristics

**Figure 1:**
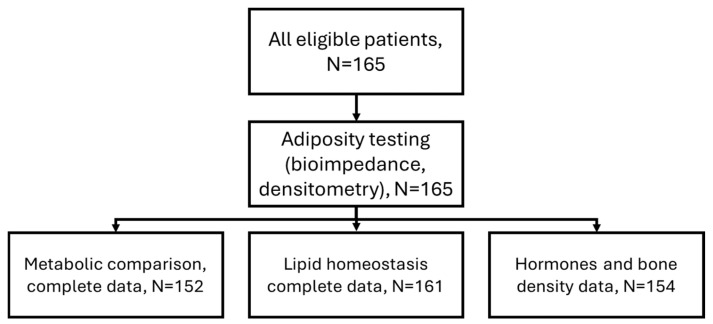
Patient flowchart.

**Figure 2:**
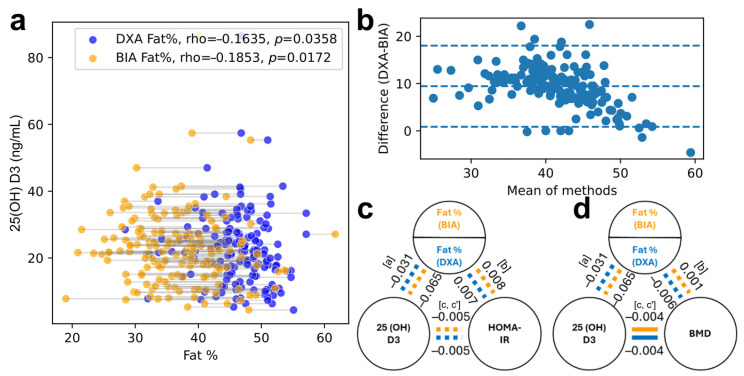
(**a**) Scatterplot of the 25(OH)D versus the adiposity measured by DXA (blue) and BIA (orange), showing similar modest inverse correlations for both modalities (Spearman rho ≈ −0.16 for DXA; rho ≈ −0.19 for BIA). (**b**) Bland–Altman plot demonstrating a systematic bias between two methods. (**c**,**d**) Mediation path diagrams illustrating the regression framework used to test whether the adiposity attenuates associations between the 25(OH)D and (**c**) the HOMA IR or (**d**) the BMD. Paths: [a] 25(OH)D → adiposity, [b] adiposity → outcome adjusted for 25(OH)D, [c] total 25(OH)D → outcome, and [c′] direct 25(OH)D → outcome adjusted for the adiposity. Dotted lines indicate non-significant paths.

Of the 165 eligible children, all had complete DXA and BIA data; specific comparisons were performed on smaller cohorts, as detailed in Figure 1. The cohort was predominantly male (52.7%), with a median age of 13 years (11–15) and a median serum 25(OH)D concentration of 21.9 (15.9–27.6) ng/mL. DXA reported higher fat percentages than BIA (median 46.6% vs. mean 36.7 ± 7.0%, Table 1). Paired plots showed close within-subject correspondence in the direction of the adiposity–vitamin D associations (Figure 2a).

### 3.2. Does the Adiposity–Vitamin D Signal Depend on Modality?

In univariate analyses, the 25(OH)D was inversely correlated with the adiposity for both modalities (Figure 2a, rho ≈ −0.16 for DXA; rho ≈ −0.19 for BIA). However, after adjusting for age, sex, and season, the adiposity was not a significant predictor of the 25(OH)D across modalities (*p* > 0.16). Season and age were the dominant determinants of the 25(OH)D, with approximately 8 ng/mL lower concentrations in winter and spring (vs. autumn) and a modest decline with increasing age.

The DXA- and BIA-estimated adiposity showed a strong linear association (R^2^ = 0.610). Although the two methods are highly correlated, they are not interchangeable across the full range of the adiposity. Agreement between the DXA and the bioimpedance adiposity estimates was evaluated using Bland–Altman analysis (Figure 2b). Among the 165 children, the mean difference (DXA–BIA) was 9.44 percentage points (95% CI 8.77 to 10.12). The 95% limits of agreement ranged from 0.86 (−0.25–2.01)% to 18.03 (16.87–19.18)% points, indicating that, for most individuals, the DXA values lay between approximately 1 and 18 percentage points above the BIA values. The distribution of the differences showed mild deviation from normality (Shapiro–Wilk *p* = 0.02), and regression of the differences on the pairwise mean demonstrated a statistically significant proportional bias, with larger discrepancies at lower adiposity levels. No sensitivity analysis was applicable.

Neither the HOMA-IR nor the atherogenic lipid fractions (triglycerides, LDL) showed consistent associations with the 25(OH)D, and the adiposity did not mediate any putative vitamin D effects with either modality (Figure 2c,d). Adiposity showed a small, similar positive association with the LDL (trend-level significance) in both the DXA-based and the BIA-based models.

**Figure 3:**
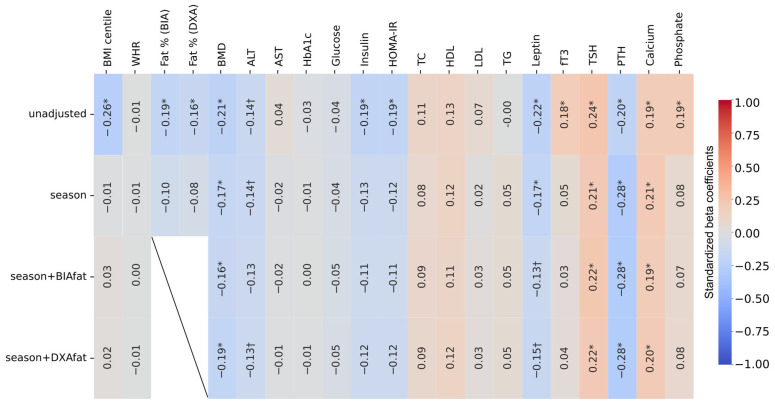
Heatmap of univariate and multivariate partial correlations with 25-hydroxyvitamin D. Asterisks mark statistical significance (* < 0.05; † < 0.10). ALT—alanine transaminase, AST—aspartate transferase, BIAfat—adiposity from bioelectrical impedance analysis, BMD—bone mineral density, BMI—body mass index, DXAfat—adiposity from dual-energy X-ray absorptiometry, fT3—free triiodothyronine, HbA1c—glycated hemoglobin A1c, HDL—high-density lipoprotein cholesterol, HOMA-IR—homeostasis model assessment of insulin resistance, LDL—low-density lipoprotein cholesterol, PTH—parathyroid hormone, TC—total cholesterol, TG—triglycerides, TSH—thyroid-stimulating hormone, WHR—waist-hip ratio.

Across more than fifty side-by-side models, physiologic conclusions were indistinguishable between DXA and BIA. Although BIA systematically underestimated fat percentage relative to DXA, this bias did not affect statistical inferences regarding vitamin D or endocrine or metabolic relationships. Where minor differences appeared (e.g., a slightly stronger adiposity–LDL slope with BIA), they did not alter the interpretation (Figure 3). No specific sensitivity analysis was applicable.

## 4. Discussion

A steady increase in the prevalence of obesity in the developmental ages has been observed in many countries, from China to Eastern Europe [3]. Our studies assessing the nutritional status of school-age children are consistent with this trend. One in five school-age children was overweight [27].

Obesity can lead to numerous adverse health changes such as insulin resistance, diabetes, dyslipidemia, hypertension, and increased cardiovascular risk, autoimmune thyroiditis, and polycystic ovary syndrome. Most obese children and adolescents also have a vitamin D deficiency, which in obesity can exacerbate complications resulting from both its calcitropic and pleiotropic effects [3,9,10,28,29].

In the group of obese patients we evaluated, a vitamin D deficiency was found in 103/165 children (62.4%) during the winter–spring period. The remaining study participants also did not reach the recommended level [9,11]. However, this did not affect the analysis of the fat content using the BIA and the DXA methods. Similar results regarding VDD in obese children have been published by other authors [3,10,28,30,31].

This study leveraged the serum 25-hydroxyvitamin D as a physiologic signal to evaluate whether dual-energy X-ray absorptiometry (DXA) provides superior insight into an adiposity-related biology compared with bioimpedance analysis (BIA) in pediatric obesity. Despite DXA being a reference standard for body composition, the present findings demonstrate that DXA and BIA yield essentially identical physiologic inferences across endocrine, mineral, and metabolic domains.

First, the adiposity–vitamin D association did not depend on modality. DXA and BIA each showed the expected modest inverse correlation with the 25(OH)D univariately, consistent with the population-level evidence linking higher adiposity to lower vitamin D [12,13,14,15,16]. However, once the season and age factors were accounted for—two dominant determinants of the vitamin D level in pediatric cohorts—adiposity, as measured by either method, was not a significant predictor. This suggests that, within a uniformly obese population, the physiologic pathways contributing to the lower vitamin D levels may already be maximally engaged, so that incremental variability in a fat percentage adds little additional explanatory value. Notably, both modalities yielded equivalent statistical inferences, suggesting that DXA does not provide an advantage over BIA for detecting adiposity-related variation in the 25(OH)D within established obesity.

In our studies of the obese children with a vitamin D deficiency, we did not find a statistically significant negative effect on the calcium and phosphate metabolism. Physiological balance was observed, as in the studies by Dura-Trave et al., who showed that the lower vitamin D concentrations were associated with higher PTH values [28]. Zhu et al. also evaluated the calcium and phosphate metabolism in obese school-age children. They observed lower calcium concentrations, similar to our group, as well as low alkaline phosphatase activity and an impact on the bone age, which was not evaluated in our group [31].

Predictors of the vitamin D deficiency in obese children compared to their peers with normal vitamin D concentrations in Xu’s study also included lower calcium concentrations, elevated ALP levels, and delayed bone age [30].

Second, the vitamin D–PTH–calcium endocrine axis was strong, consistent, and modality-independent. Lower vitamin D robustly predicted higher parathyroid hormone (PTH) levels and modest reductions in serum calcium in both the DXA- and the BIA-based models. The magnitude and direction of these associations mirrored established physiology and prior observations in pediatric obesity [23,24,25]. Notably, neither method independently predicted the PTH nor calcium, confirming that the vitamin D status—not adiposity—is the primary determinant of the PTH elevation in this cohort. Bone mineral density was not adversely associated with the vitamin D, aligning with literature showing relative preservation or elevation of the BMD in pediatric obesity due to the mechanical loading and adipokine-related pathways [26]. Again, DXA and BIA produced interchangeable conclusions.

Third, no vitamin D–metabolic signal emerged, and the adiposity did not mediate any associations with the HOMA-IR, triglycerides, or the LDL-cholesterol. This was consistent across all models. These results are in agreement with the evidence that many vitamin D–metabolic associations attenuate after the adjustment for adiposity [19,20,21,22] and with the Mendelian randomization studies indicating that the vitamin D is unlikely to play a causal role in the insulin resistance or dyslipidemia [32,33]. The small trend-level relationship between the adiposity and the LDL was similar for both modalities, reinforcing that neither method uncovers metabolic effects that the other obscures. Collectively, these findings confirm that DXA does not reveal hidden vitamin D–metabolic pathways that BIA fails to detect.

Body composition was assessed in the analyzed group of children using BIA and DXA because obese children were referred to the specialist care, and the methods used were employed to monitor the treatment. From a methodological perspective, these results highlight that although BIA systematically underestimates fat percentage relative to DXA at the absolute level, this bias does not propagate into different statistical or physiologic interpretations. Across more than fifty parallel models, DXA never produced a materially different inference than BIA. Therefore, for studies and clinical workflows concerned primarily with the physiologic inference rather than the precise body fat quantification, BIA offers a practical, scalable, and statistically equivalent alternative to DXA.

Clinically, these findings emphasize the need to interpret a vitamin D status in pediatric obesity primarily through the lenses of seasonal variation and age, rather than the incremental differences in fat mass. Vitamin D should be evaluated and treated for its well-established role in the calcium–PTH regulation, but clinicians should be cautious about expecting improvements in insulin resistance or lipid profiles through a vitamin D repletion alone. Weight management remains the principal strategy for reducing the metabolic risk, with vitamin D playing a supportive, but not mechanistic role.

This study has several limitations. Its cross-sectional design precludes causal conclusions and prevents assessment of temporal dynamics in the vitamin D or metabolic markers. The cohort consisted exclusively of children with obesity, which reduces the adiposity variability and may obscure population-level gradients detectable across a wider BMI spectrum. In population-based or mixed BMI cohorts, greater variability in the adiposity may yield a stronger adiposity–25(OH)D gradient, and modality-specific measurement differences (particularly at lower adiposity levels where proportional bias can occur) could have a larger impact on the effect estimates. Our consecutive sampling reduces but does not eliminate selection bias. Generalizability may be limited to the children with established obesity in tertiary-care settings, and the results may differ in population-based samples or in younger children. UV exposure, dietary intake, and physical activity were not directly measured, and associated residual confounding is possible.

Although DXA is considered a reference standard for body composition, no four-compartment criterion method (e.g., DXA + densitometry + total body water) was available for an absolute validation. Measurement error may differ between DXA and BIA, potentially influencing the absolute fat-percentage estimates but not the physiologic inferences. The Bland–Altman differences showed mild non-normality, which may slightly affect the precision of the limits of agreement. Nonetheless, our objective was to compare the inferential equivalence, not absolute accuracy, a question that DXA and BIA were well-suited to answer.

## 5. Conclusions

In children with established obesity, DXA and BIA provide equivalent physiologic insights when modeling the vitamin D–endocrine and the vitamin D–metabolic relationships. Although DXA yields higher absolute fat-percentage values, this measurement bias does not translate into different statistical interpretations. Vitamin D shows a strong, modality-independent association with the PTH–calcium axis but demonstrates minimal relevance to the metabolic markers, and adiposity does not mediate the vitamin D–HOMA-IR or the vitamin D–lipid relationships. Season and age dominated variation in the 25(OH)D within this population. For physiologic modeling and large clinical datasets, bioimpedance is an appropriate and practical alternative to DXA, allowing broader implementation of adiposity-adjusted analyses without the loss of inferential validity.

## Figures and Tables

**Table 1:** Patient characteristics.

Characteristic	N	% (Count) Mean (SD)/Median (IQR)
Sex (Males)	165	52.7 (87)
Age [years]	165	13 (11–15)
BMI centile	165	99 (98–99.9)
Fat %—bioimpedance [%]	165	36.73 ± 6.98
Fat %—DXA scan [%]	165	46.6 (43.8–49.3)
25-OH D3 [ng/mL]	165	21.9 (15.9–27.6)
25-OH D3 [ng/mL]—Winter	53	19 (14.5–24.5)
25-OH D3 [ng/mL]—Spring	50	19.2 (11.4–25.1)
25-OH D3 [ng/mL]—Summer	27	27.1 (23.7–31.8)
25-OH D3 [ng/mL]—Autumn	35	24.9 (21.0–29.2)
Glucose [mg/dL]	165	87 (82–93)
HbA1c [%]	165	5.1 (4.7–5.4)
Insulin [mU/mL]	152	11.3 (7.67–14.62)
HOMA-IR	152	2.47 (1.73–3.36)
TC [mg/dL]	162	155 (140–177)
HDL [mg/dL]	162	46 (39.25–51)
LDL [mg/dL]	162	107.5 (94–129)
TG [mg/dL]	161	90 (62–117)
TSH [µIU/mL]	163	2.08 (1.35–2.61)
fT3 [pmol/L]	163	5.53 (4.46–7.19)
PTH [pg/mL]	161	5.51 (4.46–7.19)
Leptin [ng/mL]	154	14.84 (9.13–22.01)
Calcium [mmol/L]	164	2.47 (2.43–2.53)
Phosphate [mmol/L]	163	1.36 (1.17–1.50)

25-OH D3—25-hydroxyvitamin D3 (calcidiol; often written as 25(OH)D), BMI—body mass index, DXA—dual-energy X-ray absorptiometry, fT3—free triiodothyronine, HbA1c—glycated hemoglobin A1c, HDL—high-density lipoprotein cholesterol, HOMA-IR—homeostatic model assessment of insulin resistance, IQR—interquartile range, LDL—low-density lipoprotein cholesterol, N—count, PTH—parathyroid hormone, SD—standard deviation, TC—total cholesterol, TG—triglycerides, TSH—thyroid-stimulating hormone (thyrotropin).

## Data Availability

The original contributions presented in this study are included in the article. Further inquiries can be directed to the corresponding author.
